# Pharmacological treatment for apnoea of prematurity—the need for an individualised approach

**DOI:** 10.3389/fped.2025.1710569

**Published:** 2025-12-18

**Authors:** Odunayo A. T. Fatunla, Nishadh A. Shakir, Coen S. Zandvoort, Maria M. Cobo, John van den Anker, Caroline Hartley

**Affiliations:** 1 Department of Paediatrics, University of Oxford, Oxford, United Kingdom; 2 Medical Sciences Division, University of Oxford, Oxford, United Kingdom; 3 Colegio de Ciencias Biologicas y Ambientales, Universidad San Francisco de Quito USFQ, Quito, Ecuador; 4 Division of Clinical Pharmacology, Children’s National Hospital, Washington, DC, United States

**Keywords:** apnoea, caffeine, doxapram, preterm, aminophylline, EEG, vital signs, biomarker

## Abstract

Apnoea—the cessation of breathing—is a common condition in preterm infants due to the immaturity of their lungs, airway and brainstem respiratory drivers. Consequently, many preterm infants will receive pharmacological interventions for the treatment of apnoea. Caffeine (or in some developing countries, aminophylline or theophylline) is usually given as first-line treatment, whereas doxapram is sometimes used as an adjunct therapy. These treatments reduce apnoeas and improve neurodevelopmental outcomes. However, there is substantial variation in individual infants’ requirement for treatment (not all infants will experience apnoea nor to the same degree). Moreover, there are considerable differences in how infants respond to treatment, for example, some infants continue to experience episodes of apnoea despite treatment. Clinical guidelines for pharmacological treatment (in particular, for caffeine) are often based on the age or weight of the infant, and there are currently no biomarkers for treatment requirement or dosing. There is a need for personalised treatment for apnoea of prematurity through the identification of suitable pharmacodynamic biomarkers. Here we narratively review current knowledge of the treatment of apnoea, focusing on caffeine, aminophylline and doxapram. We propose potential pharmacodynamic biomarkers and explore avenues for future research which will enable the testing and translation of these biomarkers for use in the neonatal unit. A personalised approach for apnoea treatment is essential to mitigate the negative short and long-term effects of both apnoea and its treatment in premature infants, ensuring that treatment can be provided at the right time and with the correct dose.

## Introduction

Apnoea of prematurity (AOP) is often defined clinically as a pause in breathing of more than 20 s, or a shorter pause that is associated with cyanosis or bradycardia, in an infant born before 37 weeks' gestation (1, 2). AOP is common, affecting around 90% of infants born weighing less than 1,000 g and a quarter of infants under 2,500 g (3). Similarly, over half of infants born before 32 weeks’ gestation experience AOP (4). Apnoea rate typically increases in the first month of neonatal life, before resolving as infants develop (5).

Infants with AOP may experience acute morbidities such as cyanosis and intermittent hypoxia (6). Early case-control studies in the 1980s and 1990s showed no clear association between recurrent apnoea and poor neurodevelopmental outcomes (7, 8); however, more recent studies have demonstrated that recurrent apnoeas may have long-term consequences including neurodevelopmental impairment and risk of severe retinopathy of prematurity, likely reflecting the impact of recurrent hypoxia and suppression of brain activity during apnoeic episodes (6, 9–13). Further research is needed to disentangle the effect of apnoea on neurodevelopment compared with other sequelae of prematurity and ascertain the extent to which apnoea causally impacts on brain development (6, 14). Optimising the treatment of apnoea in premature infants is essential to prevent the short and long-term effects.

The first line pharmacological treatment of apnoeas are methylxanthines, in particular, caffeine and aminophylline. Doxapram, a respiratory stimulant, is sometimes used as a second line pharmacological treatment for infants who do not sufficiently respond to treatment with methylxanthines (15). Following the influential Caffeine for Apnoea of Prematurity (CAP) trial (16), many centres adopted a standard dosage regime for caffeine citrate of 20 mg/kg loading dose followed by 5–10 mg/kg maintenance doses (17, 18).

However, despite the widespread use and benefits of caffeine treatment for AOP, many questions remain (19). Response to caffeine varies, with apnoeas persisting in some neonates despite treatment (20), suggesting higher doses may be warranted for those infants. Even with standardised dosing, there is high variation in serum concentrations of caffeine in neonates (21), indicating that biomarkers of efficacy may be beneficial. The optimal timing for discontinuing caffeine treatment is also unclear. Caffeine is often discontinued at a post-menstrual age (PMA) of 34 weeks, especially if the baby has been apnoea-free for at least 5 days (22), however, around 10% of infants need to restart caffeine treatment after initially stopping due to substantial and recurring apnoeas (23). Extending caffeine therapy (in infants irrespective of their need for treatment) does not reduce days of hospitalisation, as demonstrated in the recent large multicentre MoCHA randomized clinical trial, although it may reduce the number of days until infants become apnoea free (24). Evidence from a single-centre also suggests that continuing caffeine post discharge is feasible (25), indicating that prolonged therapy can support respiratory function if needed. However, caffeine exposure carries potential risks, including tachycardia, agitation, and vomiting (26–33); and at higher doses of caffeine, more serious complications such as cerebellar haemorrhage, and osteopenia of prematurity (31, 34–36) have been reported. Thus, unnecessary continuation of caffeine therapy is not without risks.

This presents a clinical dilemma, requiring careful weighing of the benefits of prolonged or higher dose therapy against the risk of adverse effects. There is therefore a clear need for research on how we can individualise treatment for each patient (19). Consideration of the pharmacokinetic and pharmacodynamic factors that influence the neonatal response are needed and the identification of effective biomarkers is essential to improve treatment options. Recent advances in our understanding of neonatal respiratory control and brain development, including the identification of cortical-respiratory coupling (the coordination between brain and respiratory activity) in neonates (37), the assessment of brain development using artificial intelligence, and “big data” approaches for vital signs analysis (38) offer opportunity for the development of physiological biomarkers and make this the ideal time to progress in this field.

This narrative review, discusses the pharmacological management of AOP with methylxanthines and doxapram, focusing on the current understanding of dosing strategies and heterogeneity in responses. We will briefly discuss the mechanisms of action and pharmacokinetics of current frequently used drugs (caffeine, aminophylline and doxapram) and management practices. We will then provide key directions for future research, focusing on approaches to individualise treatment through the identification of pharmacodynamic biomarkers and biomarkers for treatment requirements. While we acknowledge that these drugs have other clinical uses, including what O’Shea et al. (39) described as the “therapeutic creep”, referring to the extension of their use beyond AOP, this review will focus exclusively on their role in the management of AOP.

## History of management and current practice

### Caffeine

Caffeine has been given to neonates for over 40 years (3), and in 1999 was shown to be as effective as theophylline whilst having fewer side effects (40). The CAP trial in 2006 marked a fundamental turning point in caffeine use. It was a large randomised controlled trial (RCT) of 2006 infants demonstrating the benefits of caffeine in treating AOP, as compared to placebo, in reducing respiratory support duration and the rate of bronchopulmonary dysplasia (16). Follow-up studies showed that caffeine also provided better long-term outcomes, including a reduced risk of cerebral palsy, cognitive impairment and neuro-disability compared with placebo (41–43). Due to this strong evidence base, caffeine is now the preferred pharmaceutical first-line treatment for AOP and is one of the most prescribed drugs in neonatology (44).

Since the CAP trial, a number of studies have investigated alternative dosing strategies including higher dosing regimens and the time at which to start treatment (19). Whilst some studies have suggested that higher dosing regimens may have better responses with little increase in side effects (45), other studies have indicated increased severe adverse events for infants given higher doses, including risk of cerebellar haemorrhage (34), and further research is needed in this area (19). Overall, studies exploring the time at which to start treatment have indicated potential benefits for early prophylactic treatment with caffeine (given within the first three days of life) (19, 46), which has been incorporated into the practice of many neonatal units. Indeed, about half to two-thirds of neonatal units administer caffeine within the first day in some countries (47, 48). Caffeine administration in the delivery room has begun to be explored (49, 50). Some trials have also investigated caffeine in older age groups: Oliphant et al. (51) demonstrated reduced intermittent hypoxaemia in late preterm infants born between 34 and 36 weeks who received caffeine (at doses of 10 mg/kg/day or 20 mg/kg/day) compared with infants who received placebo.

The current guidelines from the World Health Organization (22) recommend the use of caffeine to prevent AOP in babies born before 34 weeks of gestation. The regimen includes a loading dose of 20 mg/kg followed by a maintenance dose of 5 mg/kg/day, and discontinuing after a PMA of 34 weeks, provided the baby has been apnoea-free for at least 5 days (22). However, disparities exist in clinical practice, with different regions producing their own guidelines, leading to variations in dosing strategies and protocols (Table 1) (2, 17, 18, 22, 52–56). Among the guidelines shown in Table 1, some use GA, others weight or both, whilst serum drug levels are rarely monitored. Moreover, there is institutional variation within countries, for example, surveys conducted by Grainge et al. (47) across 92 neonatal units in the United Kingdom in 2022, and by Unal et al. (48) across 74 neonatal units in Turkey in 2024 reported differences in initiating and stopping caffeine, as well as in caffeine doses and dosing frequency. Unit preferences and variable clinical conditions of infants were some reasons adduced to the survey findings (47). These disparities highlight the limitations of the “one-size-fits-all” approach and emphasize the need for objective biomarkers to guide individualised AOP management.

**Table 1:** Overview of selected guidelines.

Drug	Therapeutic protocol	WHO (22)	European consensus (52)	ANMF consensus group (56)	AAP (2)	FMH (54)	MOH (55)	NICE (17)	Queensland Health (53)
Caffeine Citrate	Initiation criteria								
	Treatment of AOP	<37 weeks GA	<1,251 g birth weight	Not specified	Not specified	Not specified	<37 weeks GA	Not specified	Not specified
	Prevention of AOP	<34 weeks GA	<32 weeks	Not specified	–	<34 weeks; <1,500 g birth weight On Day 1 PNA	–	≤30 weeks GA; <3 days PNA	<1,250 g birth weight
	For extubation	<34 weeks GA	<1,251 g birth weight	Not specified	–	Not specified	–	–	Not specified
	Dose								
	Loading	20 mg/kg	20 mg/kg	20 mg/kg	20 mg/kg	20 mg/kg	10–20 mg/kg/dose	20 mg/kg	20–80 mg/kg once
	Maintenance	5 mg/kg/day	5–10 mg/kg/day	10 mg/kg (5–20 mg/kg) daily	5–10 mg/kg/day	10 mg/kg/day	2.5–5 mg/kg/day	5 mg/kg/day	5–20 mg/kg/day (start at 10 mg/kg/day)
	Higher maintenance doses	–	Increase by 5–8 mg/kg/day over several weeks	Not specified	–	–	–	≥20 mg/kg/day (off-label)	–
	Post operative apnoea	–	–	10 mg/kg once	–	–	–	–	–
	Dose adjustments			Consider withholding dose if HR > 180 bpm Caution in renal impairment and therapeutic hypothermia				Reduce dose in renal and hepatic impairment	Withhold if the heart rate is >180 beats/min
	Duration of maintenance								
	For AOP	6 weeks	–	–	–	–	–	–	–
	For extubation	6 days	–	–	–	–	–	–	–
	Discontinuation	PMA >34 weeks AND Event-free for ≥5 days	–	–	33–34 Weeks PMA OR No clinically significant apnoea/bradycardia events off positive pressure for 5–7 days	34 weeks PMA or 1,500 g	–	33–35 weeks PMA	–
	Drug levels monitoring	–	–	Usually not necessary Only when using high doses or when toxicity is suspected, determine on day 5 of therapy	Not contributory to management	–	–	For doses >20 mg/kg/day	Not routinely required. Conditions for requirement not specified, but sample to be taken weekly, 12 h after administration
Aminophylline or Theophylline		Recommended if caffeine is unavailable. Other details not addressed	–	–	–	If caffeine is unavailable Aminophylline Loading 6 mg/kg/dose IV/PO Maintenance 2.5 mg/kg/dose (twice daily IV/PO) Check serum levels if able.	Aminophylline Loading 5–6 mg/kg/dose IV Maintenance 1–2 mg/kg/dose (given 6 to 8 hourly IV) Theophylline Loading 5 mg/kg/dose PO Maintenance 3–6 mg/kg/day (divided every 6 to 8 h)	–	–
Doxapram		–	–	–	–	–	–	–	–

AAP, American Academy of Pediatrics; ANMF, Australasian Neonatal Medicines Formulary; AOP, Apnoea of Prematurity; GA, Gestational Age; HR, Heart Rate; PMA, Postmenstrual Age; WHO, World Health Organization; FMH, Federal Ministry of Health, Nigeria; IV, Intravenous; MOH, Ministry of Health, The Kingdom of Eswatini; NICE, National Institute for Health and Care Excellence; PMA, Post-Menstrual Age; PNA, Postnatal Age; PO, Per Oral.

–, Not addressed by the guideline; Not specified, Criteria vaguely defined in the guideline.

### Aminophylline

Globally, caffeine is not always used for a variety of reasons. The majority of infants born pre-term, are born in low-income and middle-income countries, with complications of prematurity now among the leading causes of mortality in children under 5 years of age worldwide (57). In resource-limited settings, aminophylline is often used to treat AOP as caffeine is either unavailable or prohibitively expensive (58, 59). This is despite the knowledge that caffeine has fewer side effects and requires less drug monitoring (which can be hard to achieve in resource-limited settings) (60, 61). Therefore, a global goal is to enable more countries to use caffeine by increasing the number of suppliers, helping to establish regulations, and reducing the cost of the drug (60). Nevertheless, aminophylline is still widely used today in many countries (22, 54, 55, 58, 62–64), and so it is important to include this in any discussion of the treatment of AOP.

Current guidelines, where they exist, recommend aminophylline or theophylline as alternatives to caffeine (22, 54, 55) at a loading dose of 5–6 mg/kg followed by a maintenance dose of 1–2 mg/kg given every 6 or 8 h or at 2.5 mg/kg given twice daily (54, 55). If biomarkers for treatment requirement can be identified using readily available resources (such as short recordings of vital signs) this could enable clinicians in low resource settings to prioritise treatment for those infants who will benefit, lowering costs as well as improving patient wellbeing.

### Doxapram

Doxapram is not incorporated into many current guidelines for the management of AOP and it is occasionally prescribed off-label for this purpose (28, 29). It is typically administered as a continuous intravenous infusion at a loading dose of 2.0–2.5 mg/kg over 10–15 min, followed by a maintenance dose of 0.5–2.0 mg/kg/hour, with increments or reductions at 0.5 mg/kg/hour, every 12 to 24 h (26, 28, 65).

Doxapram is currently not the first-line medication for preventing AOP as significant knowledge gaps exist regarding its role in apnoea prevention (66). Similarly, the evidence for doxapram is less conclusive due to a lack of a large RCT (15). A systematic review including four RCTs showed that, in 137 infants, doxapram reduced apnoeas compared with placebo but was no more effective than theophylline (15, 67–70). Observational studies have reported mixed findings on the short- and long-term adverse effects of treatment (15). In clinical practice, the administration of doxapram is unpredictable as it is based on the unique needs of each infant and clinical judgment, posing challenges in formulating standard protocols (26, 29). To inform guidelines on the use of doxapram and better treatment options, larger trials to investigate its safety and efficacy, such as the ongoing multicentre DOXA-trial (65) are necessary. Further research that focuses on an individualised approach will help disentangle which neonates may respond better to doxapram (26, 27, 29, 65), enabling a risk-benefit assessment of doxapram use to be conducted to determine individual patient treatment.

In summary, Table 1 highlights some knowledge gaps, particularly regarding the duration and reinitiation of caffeine therapy in complex clinical scenarios. As not all scenarios can be addressed by guidelines, some decisions inevitably rely on clinical judgment. For example, whether to restart caffeine after suspected side effects such as tachycardia in infants with cardiac comorbidities, or whether to continue caffeine treatment in those with prolonged oxygen requirements due to other comorbidities. Moreover, whilst guidelines for caffeine are the most widely available and have the largest body of evidence, current practice still varies greatly. For example, the guidelines related to age differ by up to 4 weeks when advising on prophylactic use. These uncertainties support the need for non-invasive biomarkers to support bedside decision-making.

## Mechanism of action

The respiratory control system of the brainstem drives the rate and volume of diaphragmatic contraction and thus respiration. These control centres respond to sensory inputs to ensure that adequate gas exchange is occurring to sustain the body's metabolic demands. The inputs are split into two pathways: peripheral chemoreceptors in the carotid and aortic bodies primarily respond to hypoxemia to increase respiration (71); central chemoreceptors in the ventral surface of the medulla respond to pH changes in the central nervous system due to altering levels of carbon dioxide. Central chemoreceptors control around 85% of the overall driving force of respiration (72). Peripheral and central respiratory control centres continue to develop postnatally as *in utero* breathing only occurs intermittently for growth and not for gas exchange (73). Physiological immaturity in preterm infants results in altered responses to stimuli, for example, hypercapnia in preterms increases tidal volume but not respiratory rate, which leads to apnoea and desaturations secondary to bradypnoea (6).

Methylxanthines (including caffeine and aminophylline) act as inhibitors to adenosine receptors A_1_ and A_2A_. Inhibition of these receptors alters intracellular levels of cyclic adenosine monophosphate which can alter the release of neurotransmitters (74). Specifically in AOP, methylxanthines' action as a respiratory stimulant has been linked to lowered GABA release from centres in the medulla that controls respiration due to adenosine inhibition (75–77). This removal of the inhibitory effect of GABA allows an increase in respiratory drive to occur. Caffeine also increases the sensitivity of central chemoreceptors to hypercapnia, leading to an increase in minute ventilation (78). The evidence of the effects of methylxanthines on peripheral chemoreceptors is less clear and better understanding of carotid body function in neonates is needed before the mechanism of caffeine at this site can be properly understood (78).

Doxapram increases respiratory drive by acting at control centres in the brainstem and on peripheral chemoreceptors (79). *In vitro* application of doxapram increases evoked potentials in the pre-Bötzinger complex which controls respiratory depth and rate, and in the central hypoglossus nuclei which controls motor output of respiration (80). This has also been shown in *in vivo* electrophysiology studies of feline and canine models (81). Evidence in cats demonstrates that doxapram stimulates the carotid bodies (82), and suggests a biphasic response to doxapram with lower doses acting peripherally, whereas at higher doses direct central activation is seen (83). Similarly in anaesthetised human adults, doxapram increases peripheral respiratory drive (84).

## Pharmacokinetics

The pharmacokinetics (PK) of caffeine is not dependent on the route of administration. Caffeine after oral administration has a bioavailability of almost 100%, reaching peak plasma concentrations in 30 min to 2 h (85). Caffeine is hydrophilic and distributed evenly in all body fluids without tissue accumulation (86). It is also highly lipid-soluble allowing it to cross all biological membranes, including the blood-brain barrier, leading to a similar caffeine concentration between the plasma and cerebrospinal fluid of neonates (87). The metabolism of caffeine occurs primarily in the liver. In adults, caffeine undergoes 1-, 3-, and 7-demethylation primarily via CYP1A2 to generate the biologically active metabolites theophylline, theobromine, and paraxanthine, which can then be further demethylated to monomethylxanthine (88). Dimethylxanthine or monomethylxanthine is converted to methyluric acid by xanthine oxidase, whereas paraxanthine can also undergo 8-hydroxylation or generate 5-acetylamino6-formylamino-3-methyluracil by CYP2A6 or N-acetyltransferase-2, respectively (45). However, in neonates, approximately 85% of caffeine is excreted unchanged in the urine; in adults this proportion is less than 2% (45, 89). CYP1A2 is the cytochrome P450 enzyme responsible for more than 90% of caffeine metabolism, and studies have shown that CYP1A2 expression is not evident within the first 30 days of newborns' life due to delayed ontogeny, and CYP1A2 content in liver microsomes of infants aged 1–3 months is only 10%–15% of that in adults (45). Correspondingly, the main metabolite in newborns during the first trimester of life is caffeine—demethylation metabolism gradually matures with postnatal age and acetylation is immature until at least 1 year of age (89–91).

The serum half-life of caffeine in preterm infants is more than ten times that of adults because of immature hepatic metabolism and renal excretion (92, 93). Caffeine clearance in preterm infants is influenced by various factors such as the current weight, postnatal age, gestational age, parenteral nutrition, and serum creatinine concentration (94). A recent study revealed that caffeine clearance increases with postnatal age, especially after the first week of life, leading to a reduction in plasma caffeine concentrations until around the fourth week, when clearance becomes less rapid and stabilises (95). Earlier studies found that the elimination half-life and clearance of caffeine reach adult levels at approximately 5–6 months after birth. However, a re-evaluation and validation of ontogeny functions for CYP1A2 shows an increase in relative intrinsic metabolic clearance from birth to 3 years followed by a decrease to adult values (96). Therefore, the PK process of caffeine in neonates is variable and continues to mature with development, which needs to be taken into consideration when administered.

Doxapram is less well studied and long-term effectiveness and safety data are still awaited. Doxapram undergoes hepatic metabolism primarily via the cytochrome P450 enzyme system, particularly CYP1A2 and CYP3A4 into the active metabolite keto-doxapram. Clearance of doxapram in preterm infants also varies and is influenced by gestational and postnatal age (97). Reduced absorption of doxapram after enteral dosing means that a 33% dose increase is required to maintain equal exposure compared with intravenous dosing. No PK interaction has been shown between caffeine and doxapram (95).

## The need for an individualised approach

As postnatal and postmenstrual age increase, rapid changes in metabolism lead to differences in the way an infant will respond to a drug. Moreover, differences in genetics may lead to both variance in apnoea likelihood and an individual's response to medication (98, 99). Co-morbidities and other medications may also change apnoea rate and responses to methylxanthines/doxapram. Finally, other currently unknown factors could lead to individual differences in responses. Thus, although guidelines for the prevention of apnoea relating to age or birth weight provide some structure for practice, this approach might not be ideal. Indeed, such criteria discount infants just outside the cut-off age or weight limits from early (prophylactic) treatment. Conversely, some infants who meet the criteria but do not require caffeine therapy will nevertheless receive treatment, exposing them to unnecessary side effects. Additionally, it is difficult to know what dose to give an infant or when to stop treatment. In some instances, it seems caffeine is stopped too early, as infants who had initially discontinued caffeine can develop persistent episodes of apnoea, bradycardia, and/or desaturations, necessitating caffeine recommencement (23, 25). Whilst some guidelines do incorporate clinical events, such as the absence of apnoeas and bradycardias as an indication for stopping treatment (2, 17, 22, 52–55), this relies on the clinical interpretation and documentation of events which can be subjective, especially when they are subtle or transient, and prone to oversight (100–103). By individualising treatment dosages according to the specific needs of each patient, the risk of under or over-treatment can be mitigated, thus optimising drug efficacy while minimising the risk of adverse reactions (102).

So how can we personalise pharmacological treatment for AOP? To do so, we must identify and validate specific and sensitive clinical biomarkers. These biomarkers should be easy to obtain, providing a readily available means of evaluating an infant's response to or need for therapy. This will guide individualised treatment decisions in real-time clinical practice. In the case of treatment for AOP, we propose that biomarkers fall into two categories:

- Category 1: biomarkers which identify if there is a need for pharmacological treatment (i.e., whether to start or stop treatment);
- Category 2: biomarkers which, when a drug is administered, evaluate its efficacy or side effects, and therefore enable the dose to be optimised for the individual.

**Figure 1:**
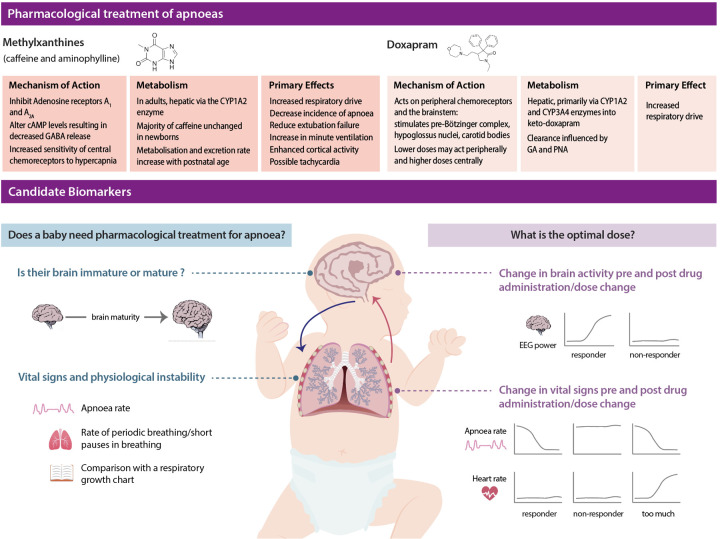
Schematic detailing pharmacological treatment of apnoea in infants and potential biomarkers. Top panel indicates the mechanism and resulting action of methyxanthines and doxapram in infants. Bottom panel highlights our proposed biomarkers for individualised pharmacological treatment of apnoea in infants. We propose that biomarkers fall into two categories. Category 1: biomarkers which identify if there is a need for pharmacological treatment (left hand side). Category 2: biomarkers which, when a drug is administered, evaluate its efficacy or side effects, and therefore enable the dose to be optimised for the individual (right hand side). EEG: electroencephalography.

How can we identify possible biomarkers? AOP relates to immaturity of the brain's control of respiration. Apnoea in turn can cause hypoxia and bradycardia. Moreover, as we have seen, pharmacological treatments for apnoea act on the brain and alter physiology. We therefore propose that metrics related to the maturity of the brain and the infant's vital signs are key candidate biomarkers for both the need for pharmacological treatment of apnoea (Category 1) and for the evaluation of drug efficacy (Category 2) (Figure 1). In the following sections we consider possible ways in which vital signs and brain activity could be used to both assess medication necessity and optimise dosing.

## Biomarkers for medication necessity (category 1)

Prophylactic use of caffeine from soon after birth is beneficial for extremely preterm infants and has been widely adopted in many centres. However, not every individual develops at the same rate. Thus, infants born near the cut-off age for prophylactic treatment may vary greatly in their respiratory and neurological development. Biomarkers to identify which infants require pharmacological treatment for AOP are essential. Such biomarkers could also be indicative of when to stop treatment. In this section, we explore in turn the possibility of using vital signs and brain activity as biomarkers for medication necessity.

Infants in the neonatal unit have their vital signs (e.g., heart rate, respiratory rate, and oxygen saturation) continuously monitored. Periods of physiological instability directly manifest in these vital signs through apnoea, oxygen desaturation and bradycardia. Thus, monitoring physiological instability using vital signs may be indicative of whether an infant needs to be administered pharmacological treatment for apnoea. Whilst this is perhaps intuitive, it may nevertheless not be as straightforward as it sounds, and we believe that infant vital signs are not currently being used to their full potential (102). Episodes of physiological instability, and in particular apnoeas, are frequently missed within clinical records (101). New approaches to identify apnoeas from vital signs recordings have been validated in infants, and so could be incorporated into monitoring (103, 104). These approaches have shown that more than 80% of apnoeas identified on vital signs recordings are missed in clinical notes (101, 103, 105). Electronic data capture of vital signs will provide an opportunity to obtain more accurate values of apnoea rates in individual infants. It will also enable the assessment of more subtle indications of whether an infant is at risk of experiencing apnoea—for example, rates of periodic breathing or short pauses in breathing could be accurately identified from electronic recordings of vital signs. Future research is needed to determine the relationship between short pauses in breathing and the likelihood of apnoea. Electronic capture of vital signs recordings will enable a detailed assessment of how respiratory dynamics vary across individuals and the relationship between respiration, heart rate and oxygen desaturation. This could be used to identify features which are predictive of the requirement for pharmacological treatment of apnoea (102).

AOP relates to immaturity of the nervous system including the brain's respiratory centres. Measures of brain maturity, and brain-respiratory interactions, may therefore provide a more detailed understanding of the development of the nervous system in an individual and their likelihood of apnoea. Recordings of electrical brain activity using electroencephalography (EEG) are not conducted routinely in every baby but can be easily performed at the cotside. EEG activity changes rapidly with the postmenstrual age of an infant. In extremely preterm infants the EEG is discontinuous, with high amplitude bursts of activity separated by periods of relative quiescence (106). As the infant ages, this activity becomes more continuous and lower in amplitude. The pattern of activity evoked by stimuli also changes with age, with older infants exhibiting spatially and temporally specific evoked responses (107–111). These types of features can be used by machine learning models to predict the so-called brain age of the infant, by comparing the features within the EEG with other infants from the model training set (110, 112, 113). This can tell us about the relative maturity of an infant's brain activity compared with their PMA. Whilst further research is needed in this area, for example, to fully address the impact of medication such as sedatives, analgesics and antiepileptics on brain age, this work holds promise to readily assess an infant's functional neurological development. By developing software to automatically assess brain age (114) and training resources for clinicians and nursing staff to accurately obtain EEG recordings, these assessments could become readily available in the neonatal unit in the near future.

Brain age gap, also known as brain age delta, can be calculated as the infant's brain age minus their PMA. Thus, it is a marker of relative brain maturity with negative values indicating that an infant has relatively immature brain activity compared with their actual age, and positive values indicate a relatively mature brain activity of the infant compared with their actual age. Very immature brain activity may be related to poor later life outcomes. However, there will also be natural variation in an infant's brain development—just as not all infants will learn to walk or talk at the same age, not all preterm infant's brain activity will develop at the same rate. We recently demonstrated, in infants studied from 31 to 36 weeks PMA, that apnoea rate is dependent on brain age gap, not postmenstrual age (115). Moreover, infants with immature brain activity had more apnoeas and desaturations in the week after they stopped caffeine compared with those with more mature brain activity, indicating that brain age may be a key candidate biomarker for caffeine requirement. Whilst these results need to be confirmed in an independent study, we believe this work offers promise for future treatment using a personalised biomarker approach.

Finally, we propose that the relationship between respiration and brain activity could be a potential biomarker for AOP medication necessity. AOP is typically attributed to immaturity of the respiratory centres in the brainstem (116). Whilst the brainstem is known to host the main respiratory drivers, it has been recently shown in adults that respiratory activity is also linked to a comprehensive network of cortical brain regions (117). This emphasises that the brain's involvement in respiration goes far beyond the brainstem. In a recent study from our research group, we examined whether this is also the case for infants. By directly relating the cortical and respiratory activity from EEG and vital signs recordings, we found that so-called cortico-respiratory coupling exists in both preterm and term infants (37). In particular, we believe the coupling we observed relates to activity in the cortical motor areas, which govern the neural control of movement (118). Since these higher-order motor areas also control lung muscles, we believe that the coupling reflects a central motor drive that provides additional coordination over lung muscles. Most strikingly, we showed that the strength of the cortico-respiratory coupling was negatively correlated with the infant's apnoea rate (i.e., a stronger coupling related to fewer apnoeas) (37). This highlights that cortical motor areas play a role in regulating AOP. Whilst this study mainly provided mechanistic insights and should be viewed as a first step, it paves the way to investigate whether cortico-respiratory coupling can serve as a biomarker, identifying which infants are at increased risk of developing AOP and therefore require treatment.

## Biomarkers to optimise dosing (category 2)

Once it has been determined that pharmacological treatment for apnoea is required, it is essential to evaluate drug efficacy and adverse effects in order to optimise the dose for an individual. Again, both vital signs and brain activity are promising candidate biomarkers to assess this since they directly underlie the physiological changes following treatment administration. Optimising dosing involves a multimodal approach to assess both response to treatment and the presence of adverse effects. For example, evaluating the presence of apnoea as well as monitoring tachycardia and agitation simultaneously will allow clinicians to better titrate the optimum dose for an individual patient (Figure 1). Some individuals will likely have clear changes in vital signs or brain activity following a dose change, whilst others may not respond. Identifying factors which can determine when an infant has reached the optimal dose or predict whether an infant will respond to a medication is an important avenue for future research. By assessing these pharmacodynamic biomarkers in real-time we will be able to balance efficacy and adverse effects to optimise the dose and treatment strategy for an individual.

Changes in caffeine dose can modulate the vital signs, for instance, by reducing the number of apnoeas (119). In the case of doxapram, vital signs have shown promising initial evidence as biomarkers to evaluate its effectiveness (26, 27, 120). In these studies, vital signs data were retrospectively analysed before and after doxapram administration. Hypoxia and the need for oxygen, quantified as the ratio between oxygen saturation (SpO_2_) and fraction of inspired oxygen (FiO_2_), accurately predicted whether treatment would be successful in an infant (27). Investigating single infants in a case series study, such composite variables distinguished between infants that did and did not respond to doxapram (26). Moreover, the data indicated that some infants may unnecessarily have started doxapram treatment whilst others were overexposed and risked adverse effects, demonstrating the clear potential for electronic recordings of vital signs rather than relying on intermittent snapshots as is often the case in clinical treatment (121). Recent findings suggested that such metrics of oxygen instability could be transferred into a bedside tool to quantify the response to doxapram treatment (120). Overall, these studies underline that vital signs may be utilised to evaluate the potential efficacy of pharmacological treatments for AOP on an individual basis and provide a framework for future work in this area.

To date, only a small number of studies have evaluated changes in EEG activity following caffeine or aminophylline administration, in particular showing an increase in EEG continuity within the first few hours after caffeine treatment has started (13, 122–125). EEG continuity quantifies the periods of ongoing “burst” activity vs. periods of inactivity—a characteristic of preterm EEG (106). An increase in continuity likely reflects methylxanthines' effect in lowering GABAergic inhibition. Doxapram may have similar effects on the EEG activity (126), though only a single study has been conducted in this area. Given the action of methylxanthines and doxapram on the central nervous system the potential for using EEG to understand the pharmacodynamic effects in infants is clear. Further research is needed to ascertain if and how the brain activity response to treatment varies in an individual, and to optimise pharmacodynamic biomarkers for use.

## Where next?

Taken together, we believe integrating continuous vital signs and EEG data could offer a comprehensive approach to optimising treatment through identifying whether infants need treatment and at what dose (Figure 1). Although studies demonstrate initial promising results, these data-driven approaches are still in their infancy. Future research is needed to increase the level of evidence for these biomarkers, consider whether they are applicable for all infants (for example, whether co-morbidities or co-medications alter the reliability), and to ensure that these tools can be easily used by clinical staff. A broader goal is that biomarkers should be able to be used globally. We acknowledge that currently in low resource settings, continuous vital sign recording and EEG monitoring are often unavailable and thus an individualised approach to treatment may be more challenging to implement. Nevertheless, if biomarkers were demonstrated to improve treatment and reduce costs in developed countries, then given the relatively low cost of EEG and vital signs monitoring they may, for example, enable allocation of caffeine (which is more costly) to the infants who will receive greatest benefit.

We believe the most important avenues for future research to expedite biomarkers for pharmacological treatment of apnoea are:

1. To identify biomarkers from the vital signs and EEG, and investigate how measures from different modalities (e.g., brain activity, respiration, heart rate etc.) relate to each other. To achieve this, initial data driven studies are needed to explore a variety of different measures.
2. To identify whether co-morbidities or other factors alter these biomarkers.
3. To test whether these biomarkers can optimise pharmacological treatment for apnoea in well-designed randomised controlled trials.
4. Finally, to facilitate adoption into the clinical environment, these biomarkers should be easily interpretable by the clinical team and straightforward to assess in individual infants. Ensuring all stakeholders are involved at every level of biomarker assessment will enable adoption into hospitals.

The biomarkers we have proposed and highlighted in this narrative review aim to assess the immediate requirement and impact of caffeine both on neurophysiology and vital signs. Nevertheless, it is important to acknowledge that other benefits of caffeine (for example on long-term neurodevelopmental outcomes) may not be fully considered with these biomarkers. Moreover, possible side effects such as agitation, feeding intolerance, and gastrointestinal disorders are not assessed. These are essential issues to consider with future research.

## Conclusion

Apnoea treatment is multifaceted, influenced by factors such as the pharmacological properties of the drug, individual genetic variability, clinical conditions, and specific clinical settings. These contribute to the variability in response to therapy among infants, making the concept of individualised therapy pertinent, in contrast to a one-size-fits-all approach that overlooks these complexities. To facilitate an individualised approach to treatment we need to identify biomarkers, both for treatment requirement and to optimise dosing in an individual. We have discussed how electronic recording of vital signs data and EEG may be used to identify such biomarkers. To date, research into methylxanthine or doxapram treatment has largely (and necessarily) focused on the benefits to groups of infants as opposed to optimising treatment in the individual. Pharmacodynamic effects using highly sampled vital signs and EEG recordings have been mostly unexplored. A shift in focus towards the individual will optimise treatment outcomes, benefitting the patient and likely saving costs. It is essential that apnoea treatment is optimised to improve the long-term outcomes of this vulnerable patient group.
